# A comparative study of RNA-seq analysis strategies

**DOI:** 10.1093/bib/bbv007

**Published:** 2015-03-18

**Authors:** Jürgen Jänes, Fengyuan Hu, Alexandra Lewin, Ernest Turro

**Keywords:** RNA-seq, transcriptome assembly, gene expression, RNA splicing

## Abstract

Three principal approaches have been proposed for inferring the set of transcripts expressed in RNA samples using RNA-seq. The simplest approach uses curated annotations, which assumes the transcripts in a sample are a subset of the transcripts listed in a curated database. A more ambitious method involves aligning reads to a reference genome and using the alignments to infer the transcript structures, possibly with the aid of a curated transcript database. The most challenging approach is to assemble reads into putative transcripts *de novo* without the aid of reference data. We have systematically assessed the properties of these three approaches through a simulation study. We have found that the sensitivity of computational transcript set estimation is severely limited. Computational approaches (both genome-guided and *de novo* assembly) produce a large number of artefacts, which are assigned large expression estimates and absorb a substantial proportion of the signal when performing expression analysis. The approach using curated annotations shows good expression correlation even when the annotations are incomplete. Furthermore, any incorrect transcripts present in a curated set do not absorb much signal, so it is preferable to have a curation set with high sensitivity than high precision. Software to simulate transcript sets, expression values and sequence reads under a wider range of parameter values and to compare sensitivity, precision and signal-to-noise ratios of different methods is freely available online (https://github.com/boboppie/RSSS) and can be expanded by interested parties to include methods other than the exemplars presented in this article.

## Introduction

Recent advances in sequencing technologies have enhanced significantly our ability to profile the transcriptomic content of cells. However, it is currently not feasible to obtain full-length (end-to-end) sequences of RNA transcripts in a high-throughput manner because high-throughput sequencers can only cope with a narrow range of complementary DNA (cDNA) fragment sizes. Thus, expression quantification is typically done via massively parallel short-read sequencing of small fragments, also known as RNA-seq. Although emergent long-read sequencing technologies are capable of producing reads matching the length of a large proportion of transcripts [1], they currently suffer from low accuracy and yield compared with established short-read technology.

There are two basic objectives that may be tackled with RNA-seq. The first is to determine precisely which regions of the genome are being transcribed in a sample. The second is to quantify the expression of these transcripts. The latter objective typically relies on completion of the former: to quantify expression of individual transcripts, a set of full-length transcript sequences is needed. This dependency is a consequence of the limited read length of RNA-seq. The requirement by high-throughput sequencers to fragment cDNA induces uncertainty in the process of reconstructing transcript sequences by assembly of short reads or alignments.

Three broad approaches have been proposed for estimating the set of transcripts in RNA samples using RNA-seq [2, 3]. The simplest approach is to assume the transcripts in a sample are a subset of the transcripts listed in a curated database, such as Ensembl [4]: reads are aligned to reference genome or transcriptome sequences, and statistical models are used to estimate expression or test for differential expression. A more ambitious strategy involves aligning reads to a reference genome and using the alignments to infer the transcript structures. Generally, maximally parsimonious solutions are preferred [5] although some methods opt for maximum sensitivity subject to constraints imposed by the alignments [6]. The most challenging approach is to assemble reads into putative transcripts *de novo* without the aid of a reference genome. The high dynamic range of expression values leads to difficulties in selecting the *k*-mer lengths for constructing de Bruijn graphs [7], while alternative splicing leads to much higher complexity in the assembly graphs than in traditional assembly of DNA-seq reads. Thus, *de novo* assembly is most fruitfully applied in the context of large-scale discovery of transcripts expressed in species with poorly characterized or absent reference genomes.

There has been a great deal of expectation that high-throughput sequencing technologies would allow a global and unbiased characterization of the transcriptome compared with microarrays and, consequently, several methods have been proposed for inferring transcript structures using sequence data. A recent assessment of a broad range of computational tools for reconstruction of transcripts from RNA-seq data has shown that low sensitivity with respect to reference annotations is a common weakness of these methods, especially as applied to eukaryotic transcriptome data [8]. However, these approaches have not been systematically compared with each other in the context of a controlled simulation study in which the true set of expressed transcripts and their expression levels are known, nor have they been compared with annotation-based inference under a range of assumptions about the accuracy of the annotations.

## Methods

We have conceived a simulation study for comparing the three approaches to expression analysis under a range of scenarios. We recall that the methods fall into three categories: those that use curated annotations to align reads and infer expression, those that use genome-guided assembly and those relying solely on *de novo* assembly of reads.

**Figure 1.**
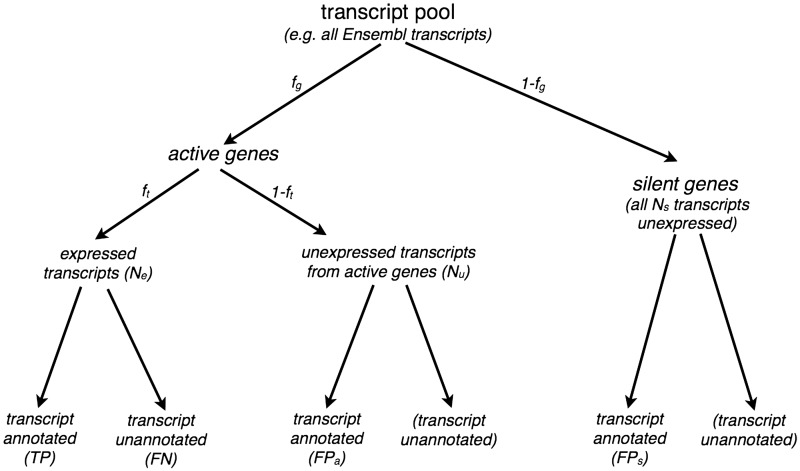
Sampling of expressed transcripts and simulated annotations from the transcript pool. We randomly designate *f_g_* of genes as active genes and leave the remaining 1 − *f_g_* as silent genes. A fraction *f_t_* of transcripts in active genes are designated as *N_e_* expressed transcripts, leaving the remaining 1 − *f_t_* as *N_u_* unexpressed transcripts (from active genes). Transcripts from silent genes are designated as *N_s_* silent transcripts. Expressed transcripts *N_e_* are assigned expression values and used as the ground truth when evaluating reconstructions. Simulated annotations of desired sensitivity *s* and precision *p* are obtained by partitioning expressed transcripts into *TP* and *FN* transcripts to reach the desired sensitivity *s*, followed by random sampling of *FP_a_* transcripts from unexpressed transcripts of active genes and *FP_s_* transcripts from silent genes to reach desired precision *p*.

The general outline of the simulation study is illustrated in Figure 1 and can be summarized as follows.

(i) Define a realistic set of transcripts as a ‘transcript pool’ and define a subset of the transcript pool as the ‘truly expressed set’.
(ii) Assign an expression value to each element of the truly expressed set and simulate RNA-seq reads accordingly.
(iii) Define the ‘discovered transcript set’ as one of
  - a set of simulated annotated transcripts that does not depend on the read data (this is a simulation of a curated annotation),
  - a set obtained through genome-guided assembly of the simulated reads or
  - a set obtained through *de novo* assembly of the simulated reads.
For each of the above, sensitivity and precision with respect to the truly expressed set can be computed.
(iv) For the transcript sets found by assembly methods, compare the discovered transcript set to the set of truly expressed transcripts.
(v) Estimate expression values for all transcripts in the discovered set.
(vi) Compare estimated and true expression values to assess the accuracy of each approach.

### Selecting the transcript pool

We select our transcript pool from the Ensembl 66 cDNA sequences. We use transcripts from subsets of the genomes throughout our study for convenience (chromosomes 21 and 22 on human, chromosomes 2 and 3 on mouse and chromosomes III and V on worm). For genome-assisted reconstruction, we use the corresponding GRCh37 reference genome. We filter out duplicate transcripts (transcripts sharing exonic coordinates and thus differing only in the locations of their untranslated regions) and alternative haplotype/supercontig entries from the cDNA sequences and the genome assembly.

### Simulating expression values

We simulate expressed transcripts by sampling from the transcript pool (Figure 1). This is close to what we would expect in reality: a large fraction of genes and transcripts are expressed only in specific tissues, developmental stages or other biological conditions. We partition the transcript pool as follows:

- Label a fraction *f_g_* of genes in the transcript pool as active and the remainder as silent.
- Label a fraction *f_t_* of transcripts belonging to active genes as ‘expressed’ and the remainder as ‘unexpressed from active genes’. We denote the number of expressed transcripts as *N_e_* and the number of unexpressed transcripts from active genes as *N_u_*.
- We denote the number of (unexpressed) transcripts from the 1 − *f_g_* fraction of genes, which are silent as *N_s_*.

**Figure 2.**
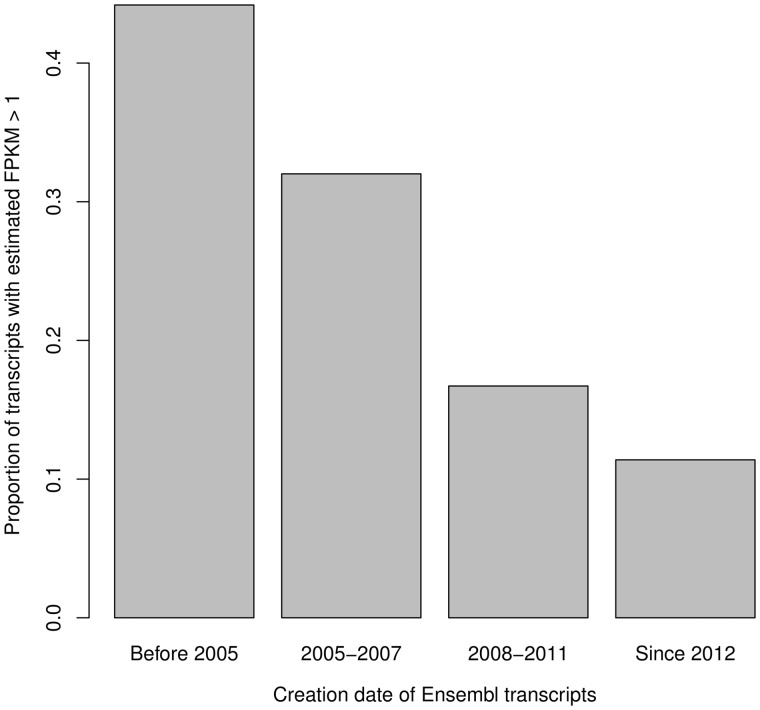
The proportion of Ensembl 70 transcripts, binned by their creation date, estimated to be moderately expressed in the thyroid sample from the Illumina BodyMap data set (accession ID ERR030872 only). The proportions are significantly different between each pair of successive bins ($p<10^{-8},\;\chi^{2}$ test for equality of proportions).

The values of *f_g_* and *f_t_* were set as described in the Supplementary Note. We hypothesized that transcripts that are often found to be highly expressed are more likely to be annotated than those that are lowly expressed or expressed only under specific conditions. We tested this hypothesis by binning transcripts by date in which they were added to the Ensembl database and examining the proportion, which were estimated to be moderately expressed (estimated FPKM > 1) in a randomly chosen sample from the Illumina BodyMap data set (http://www.ebi.ac.uk/arrayexpress/experiments/E-MTAB-513). We found that the longer a transcript had been in the database, the more likely it was to be highly expressed (Figure 2). This suggests that a realistic simulation would incorporate different sampling distributions for the expression values of annotated compared with unannotated transcripts and that this difference would depend on the quality of the annotation at a particular point in time. Naturally, such a realistic set-up would make transcript reconstruction a more challenging proposition. However, we include only the results of the more conservative analysis using a single sampling distribution, which would tend to favour reconstruction approaches. We generate artificial expression values for all expressed transcripts by sampling from a gamma distribution with shape *α* = 1.2 and rate *β* = 0.001, which captures the wide range of expression values typically found in a real sample.

### Simulating RNA-seq reads

Having generated the set of expressed transcripts and the corresponding expression values, we simulate 75 bp paired-end error-free reads. We sample the insert size (outer distance between the two paired reads) in base pairs from a normal distribution with mean *μ* = 250 and standard deviation *σ* = 30. We note that our set-up (error-free reads, a lack of sequencing biases, a single sampling distributions for transcripts designated as annotated or unannotated) generally favours reconstruction approaches and that any weaknesses in these approaches are therefore despite the use of particularly favourable assumptions. We also prefer this relatively simple set-up to more complicated simulators (e.g. [9, 10]), as it provides a neutral framework for comparing widely differing methods.

### Simulating imperfect transcript annotations

We simulate the set of curated, annotated transcripts after the expression values because we want to specify its sensitivity and precision with respect to the truly expressed transcripts. We can then compare the expression estimates of transcript sets found by assembly methods with the estimates obtained using a simulated annotation for different levels of annotation accuracy.

We randomly partition *N_e_* expressed transcripts into annotated transcripts (true positive, *TP*) and unannotated transcripts (false negative, *FN*) to achieve the desired sensitivity *s*.

$$\begin{array}{l} s=\frac{TP}{TP+FN}\\ N_{e}=TP+FN \end{array}$$

We then randomly sample *FP_a_* false positives from *N_u_* unexpressed transcripts from active genes and *FP_s_* false positives from *N_s_* unexpressed transcripts from silent genes to obtain the desired precision *p*.

$$p=\frac{TP}{TP+FP_{a}+FP_{s}}$$

The sampling of the two different kinds of false positive (FP) transcripts (*FP_a_* and *FP_s_*) is based on the assumption that the fraction of annotated transcripts is the same for both active and silent genes.

$$\frac{TP+FP_{a}}{FP_{s}}=\frac{f_{g}}{1-f_{g}}$$

This gives us four simultaneous equations, which we can solve for the four parameters *TP*, *FN*, *FP_a_* and *FP_s_*, given the required sensitivity *s* and precision *p*.

### Assessing transcript reconstruction (assembly methods only)

We compare the reconstructed/estimated transcripts to the simulated/expressed transcripts by producing a one-to-one matching between them (we describe the matching algorithm below). We proceed by classifying transcripts into three groups:

**True** **positive** transcripts are present in the estimated transcript set and can be matched to an expressed transcript.

**False** **negative** transcripts are present in expressed transcripts that cannot be matched with a transcript from the estimated transcript set.

**False** **positive** transcripts are present in the estimated transcript set and cannot be matched to an expressed transcript.

We characterize the accuracy of an estimated transcript set via sensitivity and precision. Sensitivity and precision are calculated from the labelling obtained from comparing the transcript set to expressed transcripts and using the standard definitions [sensitivity *s* = *TP*/(*TP* + *FN*), precision *p* = *TP*/(*TP* + *FP*)].

### Matching reconstructed transcripts to expressed transcripts

In principle, a one-to-one mapping of reconstructed transcripts to expressed transcripts could be achieved by finding pairs of equal transcripts between the expressed transcripts and the assembled transcripts. However, we prefer to allow flexible matching between the start and end coordinates of the assembled transcripts and the true start and end coordinates as assembly methods are known to have difficulties in recovering these exactly [11], and yet, such approximate reconstructions could still be informative and provide reasonably reliable expression estimates. On the other hand, as one of the main promises of RNA-seq is the ability to study splicing patterns, we follow [8] and require exact matching of all the exon boundaries that are not also the 5′ and 3′ ends of the transcripts.

We use the following algorithm to match reconstructed transcripts to expressed transcripts.

**Figure 3.**
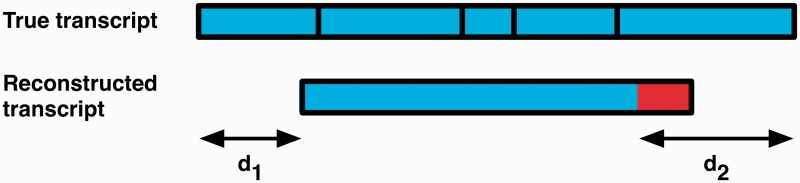
Matching reconstructed transcripts with true transcripts. The highlighted region on the right end of the reconstructed transcript indicates a mismatching sequence with the true transcript above it. Solid vertical lines indicate exon boundaries. A colour version of this figure is available at BIB online: http://bib.oxfordjournals.org.

- Create a list of ‘match candidates’ by iterating over all expressed transcripts and reconstructed transcripts. A match candidate is a pair consisting of an expressed transcript and a reconstructed transcript that fulfils the following criteria:
  - The length of the longest common subsequence of the reconstructed transcript and the true transcript is at least 80% of the length of the true transcript.
  - The longest common subsequence of the reconstruction and the true transcript contains all inner exons of the true transcript.
  - The total length of the reconstructed transcript does not differ from the total length of the true transcript by >20%.
- For every match candidate of expressed transcript *i* and reconstructed transcript *j*, we calculate a mismatch score $m_{ij}=d_{1}^{2}+d_{2}^{2}$. Here, *d*_1_ and *d*_2_ are lengths of the mismatching sequences at the two ends when transcripts have been aligned such that the inner exon sequences match (Figure 3). If several alignments are possible, we select the alignment that minimizes the mismatch score.
- We then traverse the list of match candidates sorted by increasing mismatch score. At each step, we designate the pairing (*i*, *j*) with the smallest score as a true match and, in order to obtain a one-to-one mapping between reconstructed transcripts and expressed transcripts, remove all match candidates containing the *i*-th expressed transcript or the *j*-th reconstructed transcript from the match candidate list.

### Estimating transcript expression

Once the discovered transcript set has been established through any of the three routes described above, expression estimates are obtained using MMSEQ [12]. We run MMSEQ on the entire discovered transcript set (including both TP and FP transcripts), as that is what would occur in a real RNA-seq experiment. The estimated expression values are compared with the known expression values using the matching procedure described above for the computational methods and the ‘known’ values for FP transcripts are set to zero as, by definition, FP transcripts are not present in the sample.

## Results

Here we present the results of our analysis comparing the three approaches. We selected Cufflinks to assess genome-guided assembly and Oases to assess *de novo* assembly. We limit ourselves to one method within each category to simplify our exposition and because the categories are so much more different from each other than implementations within any given category. Additionally, we assess a fourth approach, the RABT+Cufflinks method [13], which supplements a set of annotated transcripts with reconstructed transcripts using Cufflinks. Parameter settings are described in the Supplementary Note and in general have been left as default or set to recommended values based on the literature. Intricate parameter tweaking and data post-processing are beyond the scope of this work, as our objective is to compare the general properties of these methods under standard use. Unless stated otherwise, we simulate data using human transcripts as our reference.

### Reconstruction methods have low sensitivity and precision

**Figure 4.**
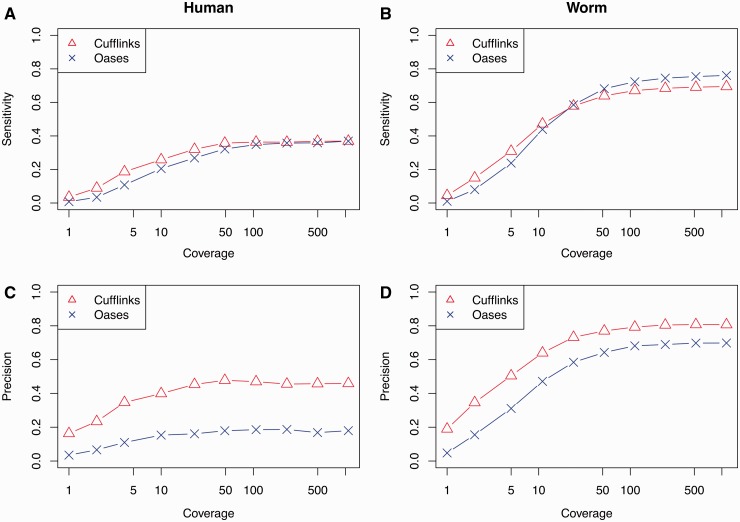
Transcriptome reconstruction accuracy for varying coverage values. Sensitivity (top panels, labelled **A** and **B**) and precision (bottom panels, labelled **C** and **D**) of transcripts reconstructed using Cufflinks (triangles) and Oases (crosses) as a function of simulated read coverage for human (left panels, labelled **A** and **C**) and worm (right panels, labelled **B** and **D**) transcriptomes. A colour version of this figure is available at BIB online: http://bib.oxfordjournals.org.

First we consider the differences between *de novo* assembly and genome-guided assembly for the reconstruction of the transcript set. We assess the sensitivity and precision achieved by these two approaches for a range of coverage values, computed as twice the number of fragments times the read length divided by the total sequence length of truly expressed transcripts. As the number of sequenced fragments increases, we observe an increase in sensitivity, which saturates at roughly *s* = 0.36 for both Cufflinks and Oases. Saturation of the number of reconstructed transcripts has been reported previously for Cufflinks assembly [14]. It was thought that saturation was reached when virtually all true transcripts had been reconstructed but we show here that saturation can be reached at low sensitivities in human (Figure 4A). These results suggest that *de novo* and genome-guided assembly have a similar ability to reconstruct truly expressed transcripts.

We also observe saturation for the precision of the estimated transcript sets (Figure 4C). Cufflinks saturates at *p* ≃ 0.45, whereas Oases saturates at *p* ≃ 0.17. This means that a significant majority of the transcripts produced by Cufflinks and Oases are false, even under very favourable simulation conditions. We stress that the false reconstructed transcripts are not technical or biological artefacts that may be found in real samples (e.g. partially transcribed RNA) as we only simulate reads from *bona fide* transcripts in our pool. The low precision observed is loosely supported by previous observations made on real data. In the Cufflinks paper [5], almost half of the constructed transcripts were unknown isoforms of known genes. In an application to a human RNA-seq data set, Cufflinks has been reported to produce a significant number of transcripts not present in Ensembl [14]. Both of these examples are based on experimental RNA-seq data, meaning that it was not possible to determine whether the ‘novel transcripts’ missing from existing annotations were true or false positives. The results presented here suggest that a large proportion of the ‘novel transcripts’ are likely to fall to the latter category.

**Figure 5.**
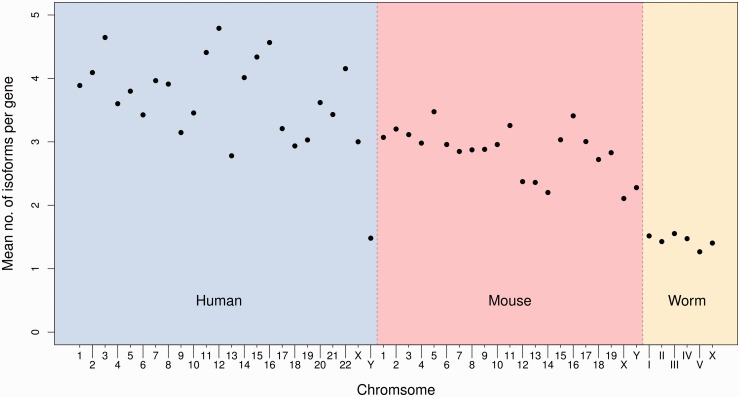
The mean number of isoforms per gene by chromosome in human, mouse and worm.

We have repeated this analysis in mouse and worm (*Caenorhabditis elegans*) to assess how reconstruction accuracy varies with transcriptome complexity. The saturation levels of both sensitivity and precision were similar in mouse compared with human for both Cufflinks and Oases (Supplementary Note). However, we see much greater accuracy in reconstructing the low-complexity worm transcriptome using Cufflinks (*s* = 0.68, *p* = 0.80) and Oases (*s* = 0.72, *p* = 0.67) (Figure 4B, D). The improved accuracy is most likely a consequence of the reduced mean number of isoforms per gene in worm compared with human and mouse (Figure 5). Reduced paralogy in the worm, as measured by the proportion of simulated read pairs mapping to multiple genes, may also play a role in this improvement (0.050, 0.023 and 0.013 for human, mouse and worm, respectively).

### Unexpressed annotated transcripts absorb a small proportion of the expression signal

Here we consider how the choice of annotation database affects expression estimation for methods that use a predefined reference transcriptome. We vary the sensitivity and precision of the simulated annotation set to assess the impact of incompleteness and inaccuracy of annotations on expression estimation. We use the simulated transcript sets to estimate expression levels and then compare the resulting expression estimates with the true (simulated) values. Our simulation set-up generates both false positives (incorrect transcripts) and false negatives (missing transcripts).

Table 1 (left) shows the correlation between the log expression estimates of TP transcripts and the true log expression values of the TP transcripts for transcript sets obtained using annotations that have a range of sensitivities and precisions. More sensitive annotations (those with a greater proportion of the truly existing transcripts) clearly lead to improved estimates. However, for a fixed sensitivity, the precision of the annotations does not have a discernible effect on the accuracy of expression estimates. This has implications for the choice of reference annotation set to be used for estimation: more sensitive annotations, such as Ensembl, which contains 190 243 (release 66) human transcripts, should be preferred over more specific annotations, such as RefSeq [15], which contains 50 029 human transcripts, if the aim is to obtain accurate expression estimates. The addition of false positives (i.e. unexpressed transcripts) into the annotation set does not have a significant impact on accuracy within the range of precision that we have considered.

**Table 1.** Performance of the simulated annotations approach

		Correlation of TPs					$\bar{\text{FP}}/\bar{\text{TP}}$				
		Sensitivity									
		0.2	0.4	0.6	0.8	1.0	0.2	0.4	0.6	0.8	1.0
Precision	0.40	**0.82** (0.88)	**0.85** (0.87)	**0.89** (0.89)	**0.93** (0.92)	**0.96** (0.96)	**0.16** (0.45)	**0.10** (0.28)	**0.06** (0.16)	**0.04** (0.08)	**0.02** (0.03)
	0.35	**0.78** (0.86)	**0.88** (0.91)	**0.89** (0.87)	**0.94** (0.92)	**0.96** (0.96)	**0.14** (0.41)	**0.10** (0.25)	**0.06** (0.15)	**0.03** (0.08)	**0.02** (0.03)
	0.30	**0.82** (0.87)	**0.89** (0.89)	**0.90** (0.90)	**0.93** (0.93)	**0.96** (0.96)	**0.14** (0.37)	**0.09** (0.19)	**0.06** (0.12)	**0.03** (0.06)	**0.02** (0.02)
	0.25	**0.81** (0.89)	**0.88** (0.91)	**0.90** (0.88)	**0.94** (0.93)	**0.96** (0.96)	**0.13** (0.31)	**0.09** (0.17)	**0.05** (0.10)	**0.03** (0.06)	**0.02** (0.02)
	0.20	**0.79** (0.86)	**0.90** (0.88)	**0.89** (0.89)	**0.94** (0.92)	**0.96** (0.96)	**0.12** (0.27)	**0.08** (0.15)	**0.05** (0.08)	**0.03** (0.04)	**0.02** (0.02)

In bold, correlation between true and estimated expression of TP transcripts (left) and $\bar{\text{FP}}/\bar{\text{TP}}$ (right) for simulated annotations of varying sensitivities and precisions (shown in the table margins). The values in brackets correspond to the correlations obtained after supplementation of transcript sets using RABT. Values shown are the mean over three independent simulations.

We also assess the propensity of different approaches to mis-assign expression signal to FP transcripts. For each method, we compute the ratio between the mean expression estimate of FP transcripts and the mean expression estimate of TP transcripts ($\bar{\text{FP}}/\bar{\text{TP}}$). A value of zero implies perfect differentiation between FP and TP expression values, whereas a ratio of 1 implies that on average FP transcripts are assigned much of the expression signal as TP transcripts (Table 1, right). Using curated annotations, $\bar{\text{FP}}/\bar{\text{TP}}$ is 0.14 on average for the lowest sensitivities we have simulated and decreases with increasing sensitivity (≤0.07 for sensitivities >0.6 irrespective of precision).

### Transcript reconstruction induces high expression estimates in FP transcripts

We now consider the performance of expression estimation using transcript sets obtained through computational reconstruction. For the two purely computationally reconstructed sets (Cufflinks and Oases), we use the transcript sets obtained at the saturated sensitivity and precision values (Table 2). These sets are obtained when mean coverage is equal to 100 and comprise the best reconstruction achievable using these methods with standard parameter settings.

**Table 2.** Performance of the computational transcriptome reconstruction methods

	Cufflinks	Oases
Sensitivity	0.36	0.36
Precision	0.45	0.17
Correlation of TPs	0.95	0.85
$\bar{\text{FP}}/\bar{\text{TP}}$	0.81	0.41

Sensitivity, precision, correlation between true and estimated expression of TP transcripts and $\bar{\text{FP}}/\bar{\text{TP}}$ for the computational transcriptome reconstruction methods.

For these transcript sets, the correlation between true and estimated expression values of TP transcripts for Cufflinks is 0.95 and for Oases is 0.85 (Table 2). A lower correlation for the *de novo* set is expected, given that the corresponding transcript set contains more non-existent transcripts, which absorb some of the true expression signal. The correlation for Cufflinks is comparable with that of some of the simulated curated annotation sets.

The $\bar{\text{FP}}/\bar{\text{TP}}$ ratio is significantly higher for computational methods than for reference-based methods using annotations with similar accuracy. This is because FP transcripts that are reconstructed from the data are necessarily supported by reads, and thus have expression signal assigned to them, whereas sequence in FP transcripts in a curated annotation set will in general not coincide with read sequence in the data, and so will be assigned low expression values. Cufflinks appears worse in this respect, as on average it assigns the equivalent of 81% of mean TP expression to FP transcripts, while for Oases this value is only 41% (Table 2). This is consistent with Cufflinks assembling fewer incorrect transcripts than Oases, which concentrates the signal in a smaller number of FP transcripts.

**Figure 6.**
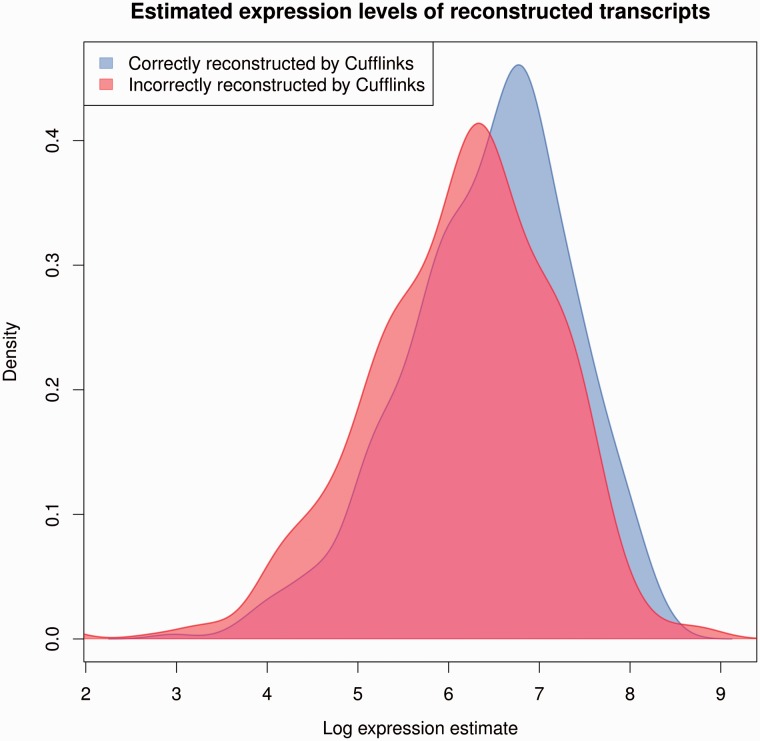
Estimated expression levels of reconstructed transcripts by Cufflinks. Densities of the log expression estimates of transcripts properly reconstructed and incorrectly reconstructed by Cufflinks ($p=2.17\times 10^{-6}$, Kolmogorov–Smirnov test). A colour version of this figure is available at BIB online: http://bib.oxfordjournals.org.

The correlation of TP transcripts reconstructed by Cufflinks is high relative to the low sensitivity (*s* = 0.36) of the method. As the more highly expressed transcripts are more likely to be reconstructed accurately (Figure 6) than the more lowly expressed transcripts and as highly expressed transcripts are easier to estimate accurately (on the logarithmic scale), the result is a high correlation for a small subset of the truly expressed transcripts.

We note that the sampling of TP annotated transcripts in our simulations does not depend on the expression level, which is favourable towards reconstruction methods. As we have shown (Figure 2), the annotated fraction of truly expressed transcripts tends to be more highly expressed than the unannotated fraction. Thus, the TP correlations for the annotation-based approach are conservative.

### Transcriptome reference-guided reconstruction provides modest improvements in accuracy of expression estimates

The Cufflinks + RABT approach supplements a curated annotation transcript set with additional reconstructed transcripts required to explain the data. In our simulation set-up, we consider the range of annotation sets with different sensitivities and precisions, as above. We present two aspects of the results: firstly the effect that RABT has on the sensitivity and precision of the final transcript set, and secondly the correlation between true and estimated expression of the TP transcripts and the overall $\bar{\text{FP}}/\bar{\text{TP}}$ ratio.

**Figure 7.**
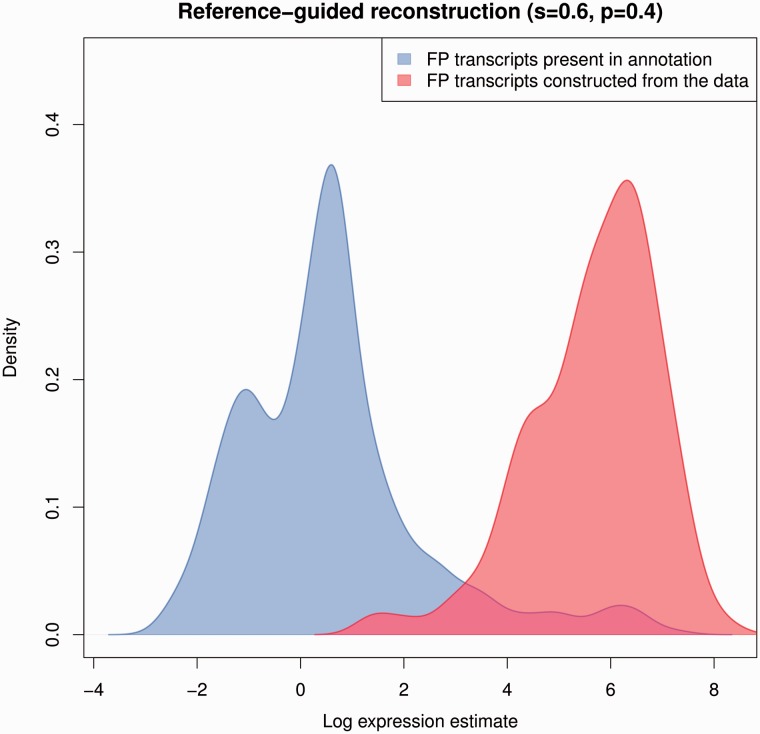
Log expression estimates for FP transcripts obtained using RABT. Densities of the log expression estimates of FP transcripts reconstructed by RABT and FP transcripts present in the simulated annotation set used by RABT as a starting point (*s* = 0.6, *p* = 0.4). A colour version of this figure is available at BIB online: http://bib.oxfordjournals.org.

Supplementing annotated transcripts with reconstructed transcripts using Cufflinks+RABT generally increases sensitivity (the starts and ends of the arrows in Figure 7 point to the annotated and supplemented sensitivities and precisions, respectively). When using annotations with the lowest sensitivity of *s* = 0.2, RABT roughly doubles sensitivity of the transcript set. The gains in sensitivity decrease substantially as the sensitivity of the annotations increases and are not noticeable beyond *s* = 0.6. Thus, Cufflinks+RABT is no better than an annotation-based approach overall when the sensitivity of annotations is moderate to high.

Surprisingly, we also observe decreases in RABT sensitivity (e.g. at *s* = 1.0 and *p* = 0.35), which is inconsistent with the Cufflinks’ manual’s statement that the RABT assembly includes ‘all reference transcripts as well as any novel genes and isoforms that are assembled’. We have found that some of these transcripts are missing from RABT output completely, whereas some are present but are missing short inner exons (and thus cannot be matched back to their true counterparts).

Table 1 shows the expression correlation between true and estimated expression for TP transcripts. There is little improvement in TP transcript correlation using RABT over using annotations alone, except at some of the lowest sensitivity values for the reference annotations.

**Figure 8.**
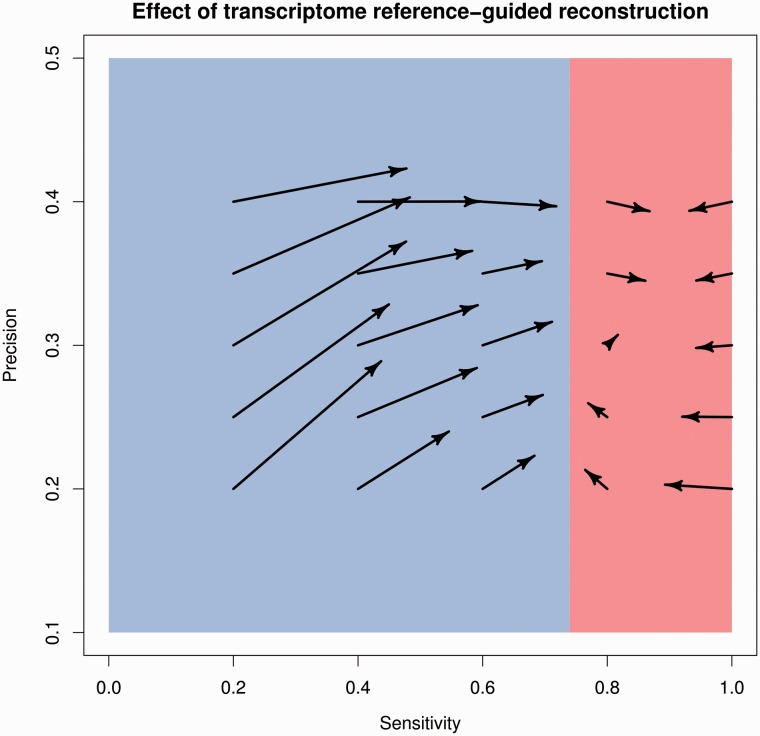
Sensitivity and precision of starting and final transcript sets obtained by RABT. Arrows start at the sensitivity and precision values of the simulated annotations and point to the final sensitivity and precision of the RABT output. Sensitivity decreases are sometimes observed when the starting sensitivity is >0.8. Values shown are the mean over three independent simulations.

RABT leads to a noticeable increase in FP expression compared with using only annotated transcripts (Table 1). This is similar to what is seen in the reconstruction methods above: by definition FP transcripts constructed by RABT are supported by reads, and so will have higher expression estimates than the FP transcripts in the annotation sets, which in general will not be supported by reads. We look at two components of FP transcripts constructed by RABT: the transcripts have a match with annotation sets and the novel ones constructed by RABT (Figure 8). Regardless of the accuracy of the reference annotation, the novel FP transcripts have higher expression estimates than the FP transcripts present in the annotation (Supplementary Note). This is consistent with the principal difference of FP transcripts coming from curated annotations and FP transcripts created by RNA-seq based transcriptome reconstruction methods: FP transcripts inferred by computational methods are (wrongly) supported by the data, making them more difficult to differentiate from TP transcripts than typical FP transcripts from curated annotations, which were derived from independent experiments.

## Discussion

We have presented a comparison of analysis approaches for RNA-seq data that use curated annotations, genome-guided reconstruction or *de novo* reconstruction. We have shown that curated annotations lead to more accurate gene expression estimates than transcriptome reconstruction methods, even when the annotations are of fairly low quality.

The main reason for this conclusion is the low sensitivity and precision of purely computational reconstruction strategies, even when assisted by a reference genome. For both *de novo* and genome-guided read assembly, our simulations show that the sensitivity of computational transcript set estimation saturates at *s* ≃ 0.36 for human RNA-seq data, meaning that many transcripts present in a sample would be missed in reconstruction—a finding that is consistent with the conclusions of [8]. The correlation between simulated and estimated expression values for the correctly reconstructed transcripts is high, but it appears that this is driven by a small number of highly expressed transcripts (see Section 3.3). Further, computational methods introduce a large number of FP transcripts (we find a precision of 0.45 for Cufflinks and 0.17 for Oases). When estimating expression, these FP transcripts absorb a substantial fraction of the overall expression signal. In contrast, even when curated annotation sets contain FP transcripts, they absorb a considerably smaller amount of the expression signal compared with computational methods (∼10% of the TP expression even at extremely low sensitivities). Curated annotations also show good expression correlation even at low sensitivities (>0.8 for an extremely low sensitivity of 0.2). The RABT approach, which supplements a curated annotation with transcripts assembled from the read data, improves sensitivity noticeably only for relatively low baseline sensitivities of the annotation set. FP transcripts introduced by RABT exhibit the same signal absorption properties as FP transcripts of pure computational methods.

Studies searching for novel elements in a transcriptome that is generally well-annotated may benefit from using a computational reconstruction or a curated annotation approach on a locus-by-locus basis. Specifically, computational reconstructions could be used in cases that strongly contradict curated annotations, such as regions with high RNA-seq counts that have no gene annotations at all and for which an approximate reconstruction would be preferred over no reconstruction at all. A straightforward implementation of this approach would be to filter from RABT output all novel transcripts that overlap known genes. Alternatively, computational predictions could be made more conservative by using reconstructed transcripts detected by several methods [11]. In analyses of data from an unannotated species without a reference genome, the use of curated annotations from a closely related species might be considered. Even if a sizeable number of transcripts differ between species, FP transcripts in the annotations would not be expected to bias expression measurements excessively.

We have restricted our exposition to a limited number of methods and animal species to assess each RNA-seq analysis strategy. We note that other *de novo* methods, such as the popular Trinity assembler [16], are becoming increasingly refined and widely used. Also, here we have not assessed the relative performance of analysis strategies in plants or bacteria, for example. We have thus made our software freely available so that new methods and species may be assessed systematically in future under a controlled simulation set-up.

Our results suggest that, despite valiant efforts, accurate estimation of the transcripts sequences in RNA samples will ultimately rely on emerging fragmentation-free sequencing technology in which read lengths can match transcript lengths. However, as the throughput of such technologies is at present still relatively low, this step may require removal of highly expressed transcripts using custom oligonucleotide capture kits [14] to ensure lowly expressed transcripts are sequenced. The use of long read technology to identify the distinct set of transcripts in a sample combined with high-throughput short read technology to estimate expression levels may be the optimal approach for the time being for accurate characterization of RNA samples.

## Supplementary data

Supplementary data are available online at http://bib.oxfordjournals.org/.

**Key Points**

- We have surveyed the three main approaches to selecting transcripts for expression estimation using RNA-seq data: reliance on database annotations, genome-guided assembly and *de novo* assembly.
- The sensitivity and precision of computational approaches—both genome-guided and *de novo* transcriptome assembly—are severely limited.
- Assembled artefactual transcripts absorb a substantial proportion of the expression signal, whereas incorrect transcripts appearing in a reference set do not absorb much signal. Thus, it is preferable to use reference sets with high sensitivity rather than with high precision. Furthermore, approaches based on annotations show good expression correlation even when annotations are incomplete.
- We have developed free software to simulate transcript sets, expression values and sequence reads under a wider range of parameter values and to compare sensitivity, precision and signal-to-noise ratios of different methods.
- Accurate estimation of the transcripts sequences in RNA samples will ultimately depend on emerging fragmentation-free long-read sequencing technology.

Supplementary Data
