# Supplementary material for: A comparative study of RNA-seq analysis strategies
Source: Brief Bioinform. 2015 Mar 18;16(6):932–40. doi: 10.1093/bib/bbv007 (PMC4652615; doi:10.1093/bib/bbv007)
Supplement: Supplementary Data [file supp_16_6_932__index.html]

A comparative study of RNA-seq analysis strategies — Supplementary Data 

# A comparative study of RNA-seq analysis strategies

## Supplementary Data

files

**Files in this Data Supplement:**

- Supplementary Data - pdf file
