# Supplementary material for: A comparative study of RNA-seq analysis strategies
Source: Brief Bioinform. 2015 Mar 18;16(6):932–40. doi: 10.1093/bib/bbv007 (PMC4652615; doi:10.1093/bib/bbv007)
Supplement: Supplementary Data [file supp_bbv007_S1.pdf]

A comparative study of RNA-seq analysis strategies

Supplementary Note

Jürgen Jänes, Fengyuan Hu, Alexandra Lewin and Ernest Turro

1 Setting f_g and f_t and simulating active transcripts

Our choice of parameter values for f_g and f_t is based on the transcripts deemed to be moderately expressed in real RNA-seq data. Transcripts with a log expression estimate exceeding -6 are labelled active. All genes having at least one active transcript are labelled active. The proportion of active genes is used to set f_g and the proportion of active isoforms out of all isoforms belonging to active genes is used to set f_t. We estimate expression using MMSEQ [1] with a reference annotation.

For human data, we use the BodyMap 2.0 dataset from Illumina ArrayExpress experiment E-MTAB-513 and Ensembl release 66 transcripts and set $f_g = 0.49$, $f_t = 0.39$.

For mouse data, we use Sanger Mouse Genomes Project whole-brain RNA-seq dataset under accession ERP000614 (`http://www.ebi.ac.uk/ena/data/view/ERP000614&display=html`). We set $f_g = 0.64$ and $f_t = 0.61$.

For worm data, we use the *C.elegans* L3-stage larval RNA-seq dataset [2] (`http://www.ncbi.nlm.nih.gov/sra/?term=srr065719`). We set $f_g = 0.4$ and $f_t = 0.86$.

The source code for obtaining these values is available from `http://github.com/boboppie/RSSS/blob/master/misc/transcript_pool_estimation`.

We recall that at least one transcript from each active gene must be active. Therefore, in order to simulate active transcripts, we first label a proportion f_g of genes as active and randomly select one active transcript from each active gene. We then randomly select additional transcripts from the remaining transcripts belonging to active genes in order to achieve the desired fraction, f_t.

2 Software versions

We use the following versions of bioinformatics software:

- python 2.7.3
- biopython 1.62
- NumPY 1.7.1
- rpy2 2.3.8
- samtools 0.1.19
- bowtie 1.1.0
- tophat 2.0.12

- cufflinks 2.2.1
- velvet 1.2.10
- oases 0.2.08
- mmseq 1.0.8a

3 Software parameter values

We use default TopHat and Cufflinks parameter values for human and mouse. However, for worm we use the values recommended in [3]:

- TopHat: `-i 30 --min-coverage-intron 30 --min-segment-intron 30`
- Cufflinks: `--min-intron-length 30`

4 Running simulations

The source code for simulations is freely available online (https://github.com/boboppie/RSSS). The main steps to run the simulation are:

- Follow the instructions in the README to download the required Ensembl datasets
- Install all the pre-required software
- Set the `RSSS_DATA_DIR` environment variable to the path containing the Ensembl data
- Execute `run_pipeline.sh` with the appropriate options: e.g. `run_pipeline.sh -c` to compute and visualise transcriptome reconstruction accuracy for varying coverage values, or `run_pipeline.sh -s` to compare sensitivity and precision values

The most up-to-date instructions are maintained on the GitHub web page above.

5 Additional results

In the main text, we present transcriptome reconstruction accuracy for varying coverage values for human and worm. Figure 1 shows the equivalent results for mouse. Sensitivity saturates at roughly 0.28 for both Cufflinks and Oases, precision saturates at around 0.42 for Cufflinks and 0.15 for Oases.

In the main text, we look at FP transcripts constructed by RABT when the reference annotation set has $s = 0.6$ and $p = 0.4$. Figure 2 shows equivalent results for a range of sensitivity and precision values.

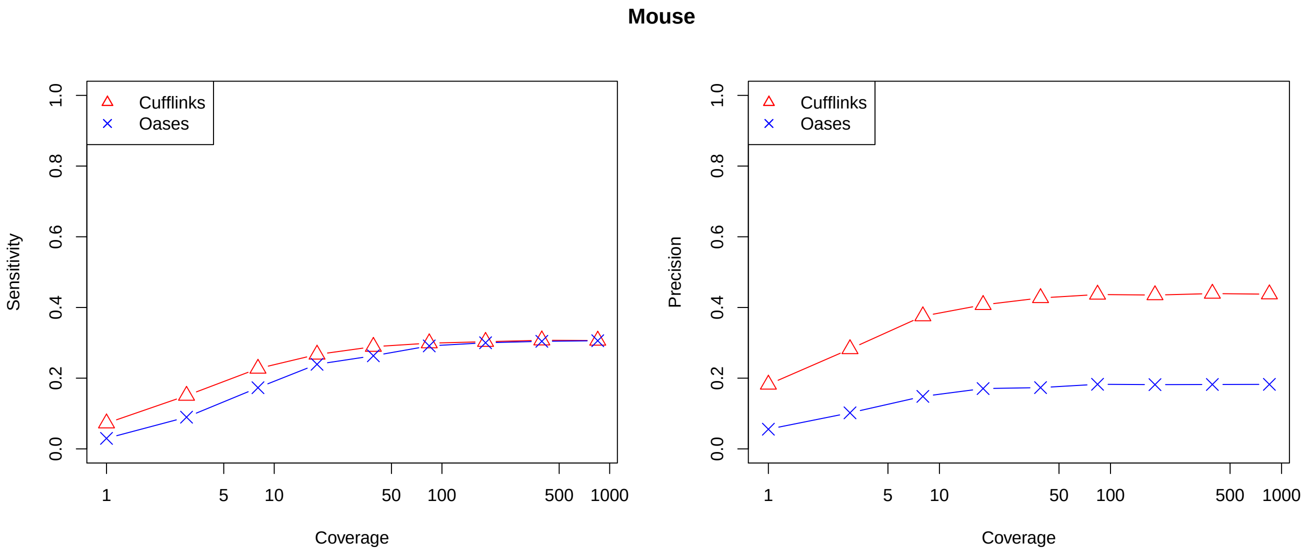

Figure 1: Sensitivity (top) and precision (bottom) of transcripts reconstructed using Cufflinks (red triangle) and Oases (blue cross) as a function of simulated read coverage for mouse data.

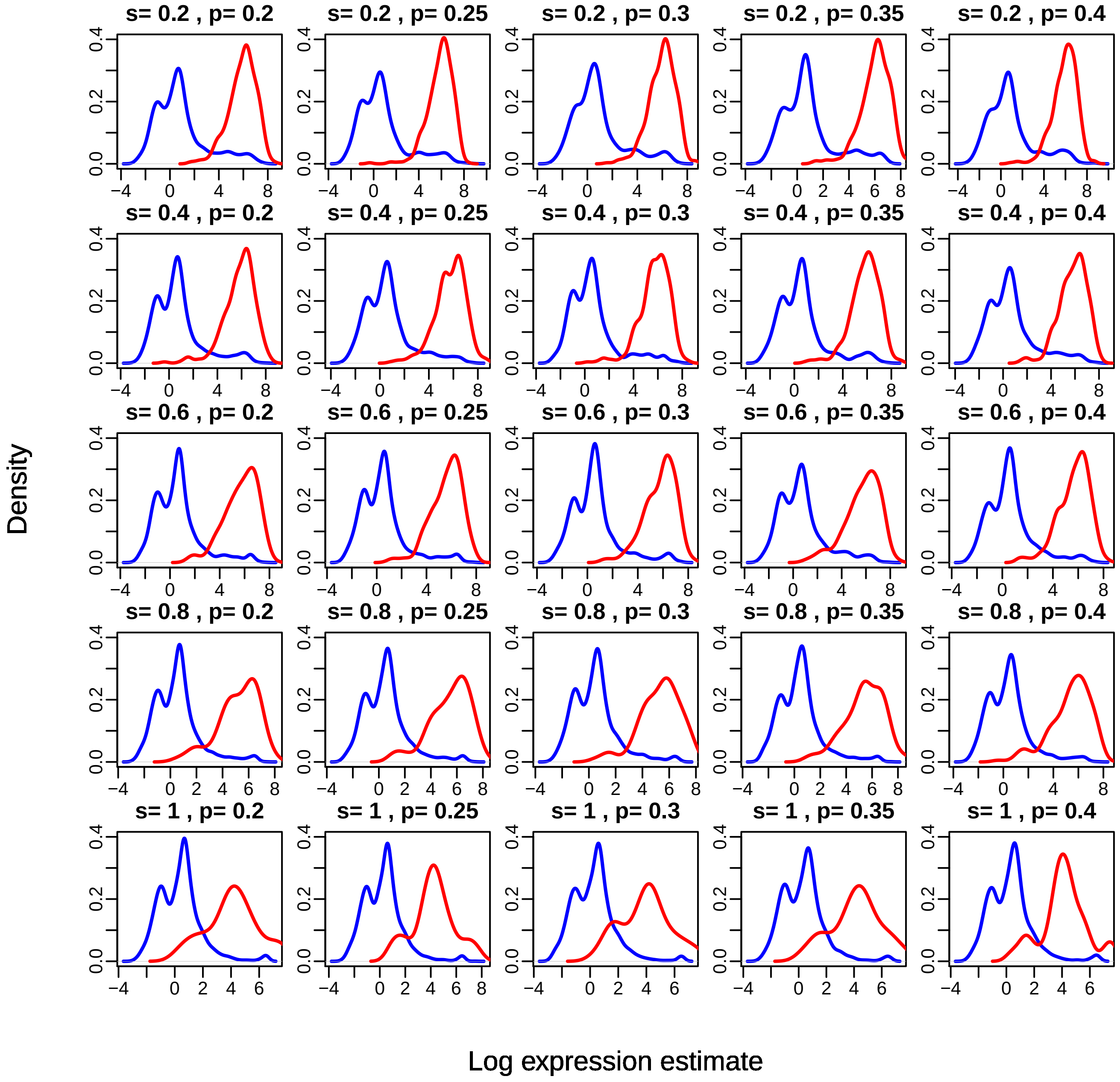

Figure 2: FP transcripts constructed by transcriptome reference-guided reconstruction in a range of sensitivities and precisions for annotation sets.

References

[1] Ernest Turro, Shu-Yi Su, Angela Goncalves, et al. "Haplotype and isoform specific expression estimation using multi-mapping RNA-seq reads". *Genome Biology* 12.2 (2011), R13.

[2] Ali Mortazavi, Erich M Schwarz, Brian Williams, et al. "Scaffolding a Caenorhabditis nematode genome with RNA-seq". *Genome research* 20.12 (2010), pp. 1740–1747.

[3] Tamara Steijger, Josep F. Abril, Pär G. Engström, et al. "Assessment of transcript reconstruction methods for RNA-seq". *Nature Methods* 10 (2013), pp. 1177–1184. ISSN: 1548-7091.
